# Effects of ionizing radiation in combination with Erufosine on T98G glioblastoma xenograft tumours: a study in NMRI nu/nu mice

**DOI:** 10.1186/1748-717X-7-172

**Published:** 2012-10-18

**Authors:** Guido Henke, Verena Meier, Lars H Lindner, Hansjörg Eibl, Michael Bamberg, Claus Belka, Wilfried Budach, Verena Jendrossek

**Affiliations:** 1 Department of Radiooncology, University Hospital Tübingen, Hoppe-Seyler-Str. 3, Tübingen, 72076, Germany; 2 Department of Medicine III, University Hospital Grosshadern, Ludwig-Maximilians-University, Marchionistr.15, München, 81377, Germany; 3 Max-Planck-Institute for Biophysical Chemistry, Am Fassberg 11, Göttingen, 37077, Germany; 4 Department of Radiooncology, University Hospital Grosshadern, Ludwig-Maximilians-University, Marchionistr.15, München, 81377, Germany; 5 Department of Radiooncology, University Hospital Düsseldorf, Moorenstrasse 5, Düsseldorf, 40225, Germany; 6 Department of Molecular Cell Biology, Institute of Cell Biology (Cancer Research), University Hospital Essen, Virchowstrasse 173, Essen, 45122, Germany

**Keywords:** Erufosine, Radiotherapy, Combination therapy, T98G glioblastoma, Xenograft tumours, Mouse model, Local control

## Abstract

**Background:**

Erufosine is a promising anticancer drug that increases the efficacy of radiotherapy in glioblastoma cell lines *in vitro*. Moreover, treatment of nude mice with repeated intraperitoneal or subcutaneous injections of Erufosine is well tolerated and yields drug concentrations in the brain tissue that are higher than the concentrations required for cytotoxic drug effects on glioblastoma cell lines *in vitro*.

**Methods:**

In the present study we aimed to evaluate the effects of a combined treatment with radiotherapy and Erufosine on growth and local control of T98G subcutaneous glioblastoma *xenograft-*tumours in NMRI nu/nu mice.

**Results:**

We show that repeated intraperitoneal injections of Erufosine resulted in a significant drug accumulation in T98G xenograft tumours on NMRI nu/nu mice. Moreover, short-term treatment with 5 intraperitoneal Erufosine injections caused a transient decrease in the growth of T98G tumours without radiotherapy. Furthermore, an increased radiation-induced growth delay of T98G xenograft tumours was observed when fractionated irradiation was combined with short-term Erufosine-treatment. However, no beneficial drug effects on fractionated radiotherapy in terms of local tumour control were observed.

**Conclusions:**

We conclude that short-term treatment with Erufosine is not sufficient to significantly improve local control in combination with radiotherapy in T98G glioblastoma xenograft tumours. Further studies are needed to evaluate efficacy of extended drug treatment schedules.

## Background

Despite recent advances in therapy of high-grade glioma the prognosis of these highly aggressive tumours is still poor. The high morbidity upon resection of the primary tumour, the early and widespread infiltration of malignant cells into the surrounding tissue, the rapid progress and the high intrinsic resistance against chemotherapy and radiotherapy are major biological factors responsible for treatment failure. Therefore, researchers aim at the identification of drugs that cross the blood–brain barrier and overcome therapy resistance for improving clinical outcome for patients suffering from high-grade glioma.

Preclinical data suggest alkylphosphocholines (APC) as promising compounds for the treatment of brain tumours, including malignant glioma: Intravenously applicable APCs like Erufosine cross the blood–brain barrier of rats and mice upon repeated parenteral administration of tolerable drug-doses [1,2] and exert potent cytotoxic effects on human glioblastoma cell lines when given alone or in combination with radiotherapy *in vitro*[3]. Agents of this drug family have a particular mechanism of action: In contrast to DNA-damaging drugs and radiotherapy, they primarily target cellular membranes thereby affecting signal transduction pathways involved in the regulation of proliferation, differentiation and survival of tumour cells [4]. Clinically relevant compounds including Miltefosine, Erufosine or Perifosine are potent inhibitors of the phosphatidylinositol-3-kinase (PI3K)/protein kinase B (Akt) pathway and the MAPK-pathway, two survival pathways that are frequently activated in high-grade glioma [5-8]. Moreover, APC induce apoptosis independently of wild type p53 [9,10] suggesting activity in p53-deficient glioblastoma cells.

Interestingly, APC increase the efficacy of chemotherapy and radiotherapy *in vitro* and in animal experiments [9]. Although the clinical use of the “first generation” APC Miltefosine (Hexadecylphosphocholine) is restricted to topical use in the setting of anticancer treatment, because of haemolytic and gastrointestinal toxicity [11], recent phase-I trials demonstrated feasibility and tolerability of pharmacologic therapy with the second generation APCs Perifosine or Erufosine alone [12-14] as well as of Perifosine in combination with chemotherapy and radiotherapy for patients with advanced human malignancies [15,16]. Of note, the combination of Perifosine with capecitabine showed a promising clinical activity in patients with metastatic colorectal cancer [16]. A potential benefit of Perifosine in combination with radiotherapy is actually being tested in patients with non-small cell lung cancer [17].

While Perifosine represents an orally applicable APC, Erufosine is the first clinically relevant intravenously applicable APC. Due to the cis-double-bond in the alkyl chain, Erufosine and the closely related Erucylphosphocholine form lamellar instead of micellar structures in aqueous solutions and therefore lack haemolytic activity. Because of modifications in the polar part of the molecule Erufosine exhibits a better solubility in aqueous solutions compared to Erucylphosphocholine thereby facilitating its parenteral injection. On the basis of its potent *in vitro* activity on glioblastoma cell lines alone and in combination with ionizing radiation, and its ability to cross the blood–brain barrier and to accumulate in the brain tissue we aimed to evaluate the effects of a combination of Erufosine and fractionated irradiation on growth and local control of T98G glioblastoma *xenograft-*tumours in immunodeficient mice. The T98G glioblastoma cell line was selected as experimental model because these highly apoptosis-resistant cells were particularly sensitive to Erufosine-treatment alone and in combination with radiotherapy *in vitro*[3].

## Methods

### Animals, cells, and tumour model

Animal experiments were conducted according to German animal welfare regulations and approved by the local authorities (registration number RO 1/05). Immunodeficient NMRI-(nu/nu)-nude mice were purchased from the University Hospital Essen (age 6–12 weeks). Animals were housed in an individually ventilated cage rack system (Techniplast, Italy). They were fed with sterile high calorie laboratory food (Sniff, Germany). Drinking water was supplemented with chlorotetracycline and potassium sorbate acidified to a pH of 3.0 with hydrochloric acid and was provided *ad libitum*.

Xenograft tumours of the human glioblastoma cell line T98G (ATCC, Bethesda, Maryland, USA) were generated by injection of 3×10^6^ cells in 300 μl medium (2+1 mixture of RPMI1640/10% FCS with matrigel (BD Biosciences)) into the axilla and serial passage of the resulting tumours. To obtain optimal suppression of residual immune activity in the donor mice a whole-body-irradiation with 4 Gy was performed 2 days before injection of the tumour cell suspension and before the first serial passage. For the experiments a source tumour was excised and tumour pieces of about 2 mm diameter were transplanted subcutaneously into the right hind limb of ether-anaesthetized mice (approximately 70% engraftment rate).

For the determination of tumour growth, tumour size was quantified with calipers in two perpendicular diameters twice a week. The tumour volume (V) was calculated as V = (a × b^2^)/2, where a and b are the long tumour axis and the short tumour axis, respectively. Body weight was monitored twice a week except for the treatment time; here, the body weight was recorded daily. Mice were randomly allocated to the treatment groups when the tumour volume reached 80–100 mm^3^. In the present experiments the median volume doubling time from the inclusion volume was 6.5 days (5.6; 8.6 d).

### Pharmacological treatment

Erufosine (ErPC3, MG 504.7) is the (N,N,N-trimethyl)-propylammoniumester of erucyl-phosphoric acid. It was kindly provided by H. Eibl, Max Planck Institute of Biophysical Chemistry, Göttingen, Germany. For aqueous solutions Erufosine was dissolved at 60°C in a mixture of destilled water and 1.2-Propandiol (Merk, Germany; mixture 98:2) to a final concentration of 24 mg/ml Erufosine and stored at 5°C after sterile filtration. For intraperitoneal or subcutaneous injection this stock solution was diluted with 0.9% sodium-chlorid solution in the appropriate ratio to obtain the required Erufosine-concentration in 100 μl for a body weight of 30 g. Difference in body weight of the mice were adjusted with injection volume.

To establish the schedule of Erufosine-treatment for the present study we analyzed drug-accumulation in *xenograft* tumours in preliminary experiments. Eight intraperitoneal injections of Erufosine every 48h at doses of 20 mg/kg BW or 40 mg/kg BW yielded tumour concentrations of 197 ± 52 nmol/g or 626 ± 76 nmol/g, respectively (means ± SD; n=4) at the end of Erufosine-treatment. Such concentrations are above the drug concentrations (50 μMol/l Erufosine equal to a tissue concentration of 50 nmol/g) known to potently induce tumour cell death when given as single drug and for increasing the cytotoxic efficacy of ionizing radiation *in vitro*[3].

Accordingly, in the experimental study mice were treated by eight intraperitoneal injections of 40 mg/kg body weight (BW) Erufosine to a cumulative dose of 320 mg/kg, respectively. Intraperitoneal injection was used as standard administration route because we showed in our earlier investigation that intraperitoneal injection was generally well tolerated whereas repeated subcutaneous injections of high-dose Erufosine regularly caused local reactions at the injection site at similar bioavailability although less gastrointestinal side effects were observed [1]. If a weight loss between 5-10% was detected, treatment was continued by subcutaneous drug administration (21% of injections). If a weight loss of above 10% was detected upon subcutaneous injection or animals appeared to suffer (immobility, loss of appetite, pale skin, retraction of the abdomen), drug treatment was discontinued until recovery and animals were evaluated as “*intent to treat*” (1% of injections). The descision was taken by the responsible scientist (G.H) on the basis of weight measurement and control of the animal behavior before each injection. Control animals received an intraperitoneal injection of the solvent.

### Analysis of ErPC3 in tumour tissues

For the quantification of Erufosine in plasma and tissue samples liquid chromatography-tandem mass spectrometry (LC-MS/MS) was employed with a deuterium labelled analogue (ErPC3-D9, MW 512.82) as internal standard as described in detail elsewhere [14].

For the analysis of Erufosine-concentrations in subcutaneous xenograft tumours, the tumours were removed after diethylether anaesthesia and immediate cervical distortion 1 day after the end of the Erufosine treatment. The tumours were weighed and stored at −20°C until analysis. The Erufosine-concentration was then analyzed using the frozen tissue samples.

### Irradiation treatment, follow up, determination of tumour growth delay and TCD50 values

Fractionated irradiation was given under ambient conditions with a linear accelerator (6 MV photons, 400cGy min^-1^ dose rate) to the tumour on the right hind limb of the animals as described earlier [18]. Shielding reduced the dose to the animal body to less then 3% of the prescribed tumour dose.

**Figure 1:**
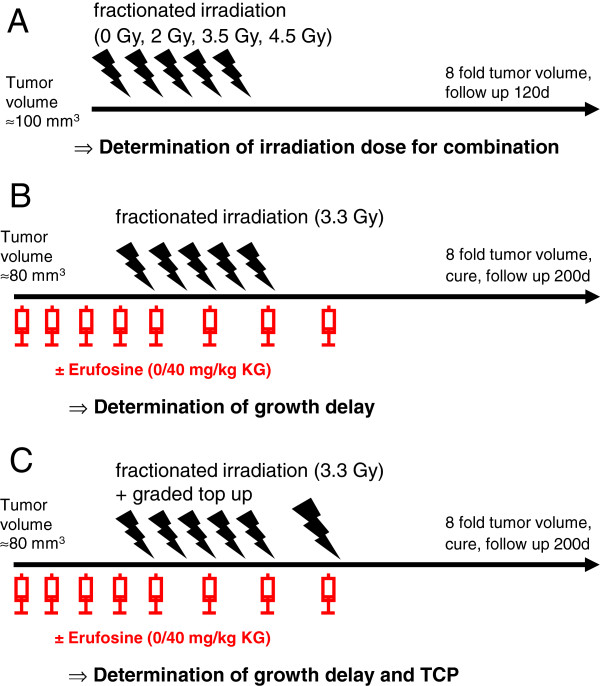
**Experimental design.A** Determination of single dose for fractionated irradiation in combination therapy (Exp. 1). **B** Determination of growth delay (Exp. 2). **C** Determination of growth delay and tumour control probability (TCP; Exp. 3).

The study was performed in three different experimental set-ups (Figure 1):

To estimate the irradiation dose for the combination experiments animals were randomized to 4 treatment groups receiving no irradiation (n=7), fractionated irradiation of 5x 2 Gy (n=8), 5x 3.5 Gy (n=7) and 5x 4.5 Gy (n=7) alone (Experiment 1; Figure 1A). The median tumour volume in this experiment at start of treatment was 105 mm^3^ (98; 141 mm^3^). Follow up was discontinued after 120 days from treatment, in case of intercurrent death or if the volume of the progressive or recurrent tumours reached 8-fold the starting volume. An exponential regression model was used to interpolate a modified growth delay endpoint as time after onset of irradiation treatment needed to achieve 4- and 8-fold size of the initial tumour volume at the start of treatment for each single tumour. Animals that appeared to suffer were killed before reaching these endpoints and were considered as censored at this time-point.

In experiment 2 (Figure 1B), fractionated irradiation with a fixed radiation dose of 5 × 3.3 Gy was applied in combination with eight injections of 40 mg/kg BW Erufosine. This Erufosine-dose had been shown earlier to yield tissue concentrations sufficient to increase the efficacy of ionizing radiation *in vitro*[1]. Erufosine was injected daily beginning 5 days prior to irradiation to allow appropriate accumulation of the drug in the tumour tissue at the onset of radiotherapy followed by 3 injections of Erufosine every 48h without or with concomitant fractionated irradiation. Animals were randomized to the following treatment groups: i) no irradiation/no Erufosine (n=18), ii) fractionated irradiation/no Erufosine (n=14), iii) no irradiation/Erufosine (n=21), iv) fractionated irradiation/Erufosine (n=20). Because of the pre-treatment with Erufosine, mice were recruited to the experiment with a smaller median tumour volume of 83 mm^3^ (81; 91 mm^3^) compared to the first experiment in order to achieve comparable tumour volumes at the onset of irradiation treatment on day 5. Follow-up was discontinued after 200 days from treatment of the last set of animals, in case of intercurrent death or if the volume of the progressive or recurrent tumours reached 8-fold the starting volume. Growth delay after the onset of Erufosine treatment was determined as described above.

Volume doubling times (VDT) were defined as time interval between the start of treatment to the day when the tumour reached twice the initial tumour volume in case of continuously growing tumours. If a transient regression occurred upon treatment the volume doubling time was calculated as interval from the first day of increase of tumour volume to the day when the tumour reached twice the minimal tumour volume.

Experiment 3 was designed to determine the tumour control probability. For this, a graded top irradiation - estimated to achieve local tumour control with the higest top-up dose from experiment 1 - was added after fractionated irradiations (5 × 3.3 Gy) with and without 40 mg/kg BW Erufosine (Figure 1C). The median tumour volume in this experiment at start of treatment was 90 mm^3^ (87; 108 mm^3^). Follow up was similar to the procedure described above. Tumour control rates at day 200 after the end of treatment were determined with respect to censored animals as described by Walker and Suit [19]. Animals with identical treatment from the second and third experiment were pooled since no significant difference in corresponding parameters occurred.

### Statistics

For the delineation of the time course of the experiments and of tumour doubling times data are expressed as median values with the upper and lower 95%-confidence intervals as given by Sachs [20].

Comparison of tumour size or volume doubling time between groups was done by nonparametric Mann–Whitney tests in case of two groups and Kruskal-Wallis tests with Dunn’s post-tests, using GraphPad InStat 3.05 (Graph Pad Software, Inc.). P -values of p ≤ 0.05 were considered statistically significant.

Growth delay is presented according to the method of Kaplan and Meier considering achievement of the 4- and 8-fold size of the initial tumour volume as event and taking animals without the respective tumour volume at the end of the observation period or undergoing intercurrent death as censored at that time-point. Differences between groups were tested for significance by the Wilcoxon-test. Median growth delay with 95%-confidence intervals was calculated with lognormal approximation implemented in JMP 9.0.0® software (SAS institute Inc.).

For estimation of the dose dependent tumour control probabilities including 95%-confidence intervals (CI), the results of the top up dose experiments including the data for control tumours (no cures at 0 Gy) were modelled by using the logit module of a commercial software package (XLSTAT2011® by Addinsoft SARL).

Arithmetic means with standard deviation are presented and student′s t-test was applied when appropriate.

## Results

### Effects of single modality treatment on tumour growth

**Figure 2:**
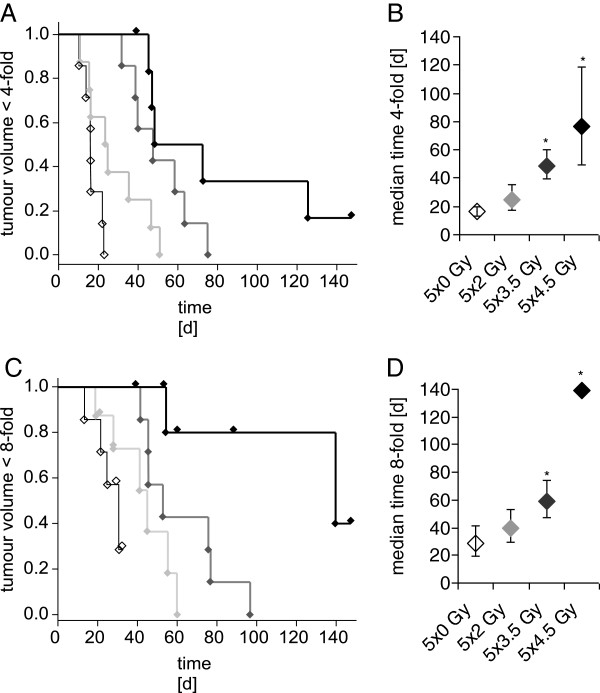
**Effects of single radiation treatment on tumour growth.A** Modified growth delay to four-fold of starting volume after fractionated irradiation. Given are Kaplan-Meier-plots of tumours after treatment with increasing doses of fractionated irradiation (open diamonds, control; grey diamonds, 5 x 2 Gy; dark grey diamonds, 5 × 3.5 Gy; black diamonds, 5 × 4.5 Gy). **B** Respective computed median growth delay with 95%-confidence interval (n=7-8), * indicates p<0.05 vs. control. **C** Modified growth delay to eight-fold of starting volume after fractionated irradiation. Given are Kaplan-Meier-plots of tumours after treatment with increasing doses of fractionated irradiation (open diamond, control; grey diamond, 5 × 2 Gy; dark grey diamond, 5 × 3.5 Gy; black diamond 5 × 4.5 Gy). **D** Respective computed median growth delay with 95%-confidence interval (n=7-8), * indicates p<0.05 vs. control.

We first established the appropriate doses of fractionated radiotherapy for the combination experiments (Figure 1A). Fractionated irradiation of the tumours led to a dose-dependent delay in tumour growth. Growth delay to the 4-fold initial tumour volume was 16, 24, 49 and 76 days for untreated controls or 5 fractions of 2 Gy, 3.5 Gy and 4.5 Gy, respectively (Figure 2A/B). The median growth delay to the 8-fold initial tumour volume was 29, 40, 59 and 139 days for the same treatment groups (Figure 2C/D). The growth delay was significant for treatment with 5 × 3.5 Gy and 5 × 4.5 Gy compared to untreated control tumours. In one out of seven animals the tumour was controlled locally upon treatment with 5 × 4.5 Gy after 120 days, whereas all other tumours recurred.

In the experimental study mice were treated by eight intraperitoneal injections to a cumulative dose of 320 mg/kg. In order to achieve an appropriate Erufosine concentration within the tumour tissue during the course of radiotherapy mice were pretreated with 5 daily injections of 40 mg/kg body weight (BW) Erufosine followed by 3 injections of Erufosine every 48h without or with concomitant fractionated irradiation (Figure 1B). To evaluate the effects of short-term treatment with Erufosine alone (5 days) the tumour volume was measured in all animals treated with 40 mg/kg BW Erufosine and all animals without Erufosine administration before the onset of fractionated irradiation. The mean relative tumour volume after 4 days of treatment with 40 mg/kg Erufosine alone compared to the starting volume (1.0) was 1.42 ± 0.39 (n=64) whereas the relative volume of the tumours in the untreated control group amounted to 1.77 ± 0.63 (n=55). These data are based on the individual increase in tumour volume and provide evidence that short-term treatment with Erufosine alone causes a significant growth-inhibition in T98G xenograft tumours (p< 0.05).

### Effects of combination therapy on tumour growth and local control rates

To detect a beneficial effect of a combined treatment with signal transduction inhibitors and radiotherapy it is important to use a radiation dose which induces a significant but moderate tumour growth delay without causing prominent local control as single treatment. On the basis of the results from Experiment 1 we selected a dose of 3.3 Gy per fraction. To gain insight into treatment-related toxicity we recorded the median relative weight of the nude mice in the four treatment groups. Radiation alone failed to induce a relevant weight loss whereas treatment with 40 mg/kg BW Erufosine alone or in combination with fractionated irradiation led to a transient median weight loss of about 10% (data not shown). Also the number of Erufosine-treated mice that died during treatment was equal in the nonirradiated and irradiated treatment groups (in total 6 out of 78 mice; 7%). These observations reveal that the addition of fractionated irradiation to the Erufosine treatment does not cause unexpected additional toxicity.

**Figure 3:**
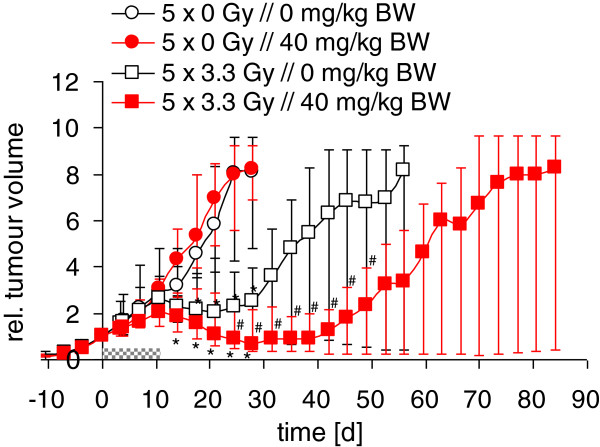
**Effects of combination treatment on relative tumour volume.** Animals received no irradiation combined with 8 injections of vehicle (open black circles, n=18) or 40 mg/kg BW Erufosine (red circles, n=21). Alternatively, animals received fractionated irradiation (5 × 3.3 Gy) combined with 8 injections of vehicle (open white squares, n=14) or 40 mg/kg BW Erufosine (red squares, n=20). Erufosine was given daily before the onset of radiotherapy and every 48h during the course of fractionated irradiation. Grey bar notifies treatment time. * indicates p<0.05 vs. control, ^#^ indicates p<0.05 vs. irradiation alone. Median relative tumour volume is given with upper and lower 95% confidence intervals.

The course of the median tumour volume in the four treatment groups is depicted in Figure 3. As expected, fractionated irradiation with 5 × 3.3 Gy led to a transient decrease in median tumour volume and a delay in tumour growth which became significant after 14 days. However, the transient early growth delay observed during short-term treatment with Erufosine alone was not maintained upon discontinuation of drug treatment in the longer follow-up and no decrease in tumour volume was detected upon Erufosine treatment without irradiation. Nevertheless, the addition of Erufosine qualitatively enhanced the decrease in tumour volume induced by radiotherapy alone, yielding significant differences when the irradiation groups were compared (Figure 3).

**Figure 4:**
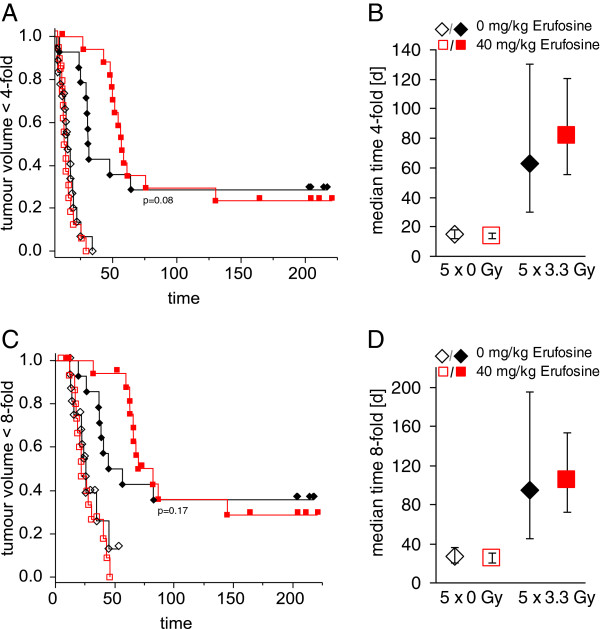
**Effect of combination treatment on tumour growth delay. A** Modified growth delay to four-fold of starting volume after combined treatment. Given are Kaplan-Meier-plots of mock-irradiated tumours combined with vehicle (open black diamonds) or 40 mg/kg BW Erufosine (open red squares) and 5 × 3.3 Gy combined with vehicle (closed black diamonds) or 40 mg/kg BW Erufosine (closed red squares). **B** Respective computed median growth delay of data obtained in A) with 95%-confidence interval (n=14-18). **C** Modified growth delay to eight-fold of starting volume after combined treatment. Given are Kaplan-Meier-plots of mock- irradiated tumours combined with vehicle (open black diamonds) or 40 mg/kg BW Erufosine (open red squares) and 5 × 3.3 Gy combined with vehicle (closed black diamonds) or 40 mg/kg BW Erufosine (closed red squares). **D** Respective computed median growth delay of data obtained in C) with 95%-confidence interval (n=14-18).

In line with these results, the median growth delay until completion of the 4- and 8-fold initial tumour volume significantly increased after fractionated irradiation. Again, laying emphasis on the early observation period, a trend (Wilcoxon-Test p=0.08) to an increased tumour growth delay was observed when Erufosine treatment was used in combination with fractionated irradiation (Figure 4). The respective growth delay to 4- and 8-fold initial tumour volumes upon Erufosine-treatment amounted to 14 and 25 days compared to 15 and 27 days in the untreated controls without irradiation and 82 and 105 days compared to 63 and 94 days in the controls with irradiation.

**Figure 5:**
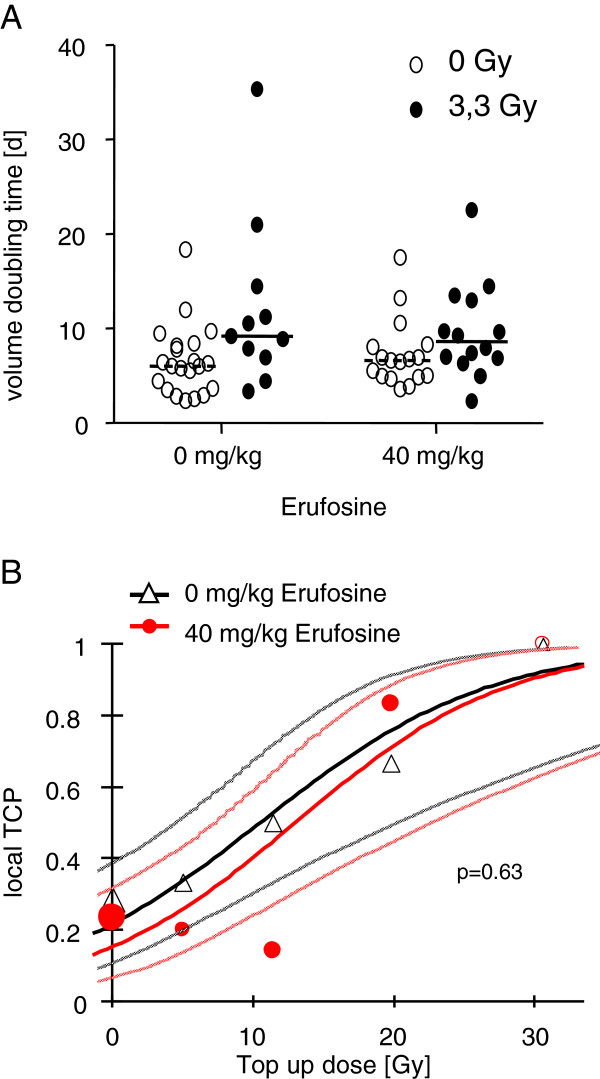
**Effect of combination treatment on volume doubling time and tumour control. A** Volume doubling time (VDT) for progression or re-growth of individual tumours after irradiation with 5 × 3.3 Gy (black circles) or without irradiation (white circles) and treatment with vehicle or 40 mg/kg BW Erufosine as indicated. Horizontal lines indicate median values of the treatment groups. **B** Local tumour control rates and calculated tumour control probabilities after fractionated irradiation with 5 × 3.3 Gy and graded top up doses (0–30.6 Gy) combined with injection of 40 mg/kg BW Erufosine (closed red circles) or vehicle (open black triangles). Thin lines indicate 95%-confidence intervals, size of symbols reflects number of animals (n=5-21).

In contrast, considering the volume doubling times of re-growing tumours, no significant differences were found when treated tumours with transiently decreased growth were compared with untreated continuously growing controls. In fact, the median volume doubling times were 6.0 (95% CI: 3.7;8.2), 6.6 (4.9;8.3), 9.2 (4.4; 21.0) and 8.6 (6.3;13.5) days for untreated controls, Erufosine alone, fractionated irradiation alone and combination therapy, respectively (Figure 5A).

In the final experimental set-up, we analyzed the tumour control rates after additional top up irradiations (Figure 1C). Single doses of 0, 5, 11.4, 19.8 and 30.6 Gy following fractionated irradiation with 5 × 3.3 Gy achieved control rates of 29%, 33%, 50%, 67% and 100% without Erufosine treatment and 24%, 20%, 14%, 83% and 100% upon Erufosine treatment (Figure 5B). The resulting TCD50 values from the calculated tumour control probabilities were 27.0 (21.1; 36.8) Gy in the absence of Erufosine and 29.5 (23.8; 39.2) Gy in the presence of Erufosine showing no significant difference. Altogether, these data reveal that the trend to increased efficacy of ionizing radiation in combination with short-time Erufosine treatment observed in the combination experiment with respect to growth delay and transient tumour volume reduction had no impact on treatment efficacy in terms of tumour control.

## Discussion

In the present investigation, we show for the first time that short-term treatment with the intravenously applicable Erufosine causes a transient decrease in the growth of the T98G glioblastoma tumours. This effect was associated with a significant accumulation of Erufosine in the xenograft T98G tumours upon repeated drug applications. Moreover, we observed a trend to an enhanced radiation-induced growth delay of T98G xenograft tumours when fractionated irradiation was combined with short-term Erufosine-treatment. However, these effects failed to translate into beneficial drug effects on fractionated radiotherapy in terms of local tumour control.

We show that repeated parenteral injections of 20 or 40 mg/kg BW Erufosine result in a significant accumulation of this membrane-targeted agent in T98G xenograft tumours. This corroborates findings of Vink and co-workers showing accumulation of the closely related compound Perifosine in KB squamous cell carcinoma xenograft tumours upon repeated oral drug administration [21]. Interestingly, short-term treatment with Erufosine caused a transient growth-inhibition within 5 days. However, the tumours resumed growth within a few days after discontinuation of drug treatment. This suggests that although the drug reaches concentrations in the tumour tissue that are sufficient for cytostatic and/or cytotoxic effects *in vivo* this treatment schedule is not sufficient to eradicate a considerable number of clonogenic tumour cells and thus, tumour stem cells. In line with our findings a recent study showed growth inhibitory effects of treatment with the closely related Perifosine in another glioma xenograft model (U251). Since the growth delay described by the authors was more pronounced compared to the results obtained in our study we speculate that the higher cumulative dose (475 mg/kg) of Perifosine upon oral administration may be responsible for the improved drug action [22]. Similarly, Li et al. [23] detected a substantial growth delay in neuroblastoma xenograft tumours in nude mice upon a 30-day treatment with Perifosine. These data suggest that *in vivo* efficacy of these compounds may depend on an extended treatment schedule.

Otherwise, the heterogeneous outcome of preclinical and clinical studies with Perifosine implicates that the efficacy of treatment with the membrane-targeted APC also largely depends on the cell type. Whereas extended treatment with Perifosine was without single drug effect in prostate cancer xenografts *in vivo*[24] and no evident clinical effect of Perifosine was seen in pancreatic cancer, squamous cell carcinoma of the head and neck, breast cancer and melanoma [25-28], the drug had clinical activity in hematological malignancies [29] and to some extent in soft tissue sarcoma or prostate cancer [30,31].

We also found that T98G xenograft tumours respond to fractionated irradiation with a dose-dependent tumour growth delay. Already with doses of 5 × 3.5 Gy (17.5 Gy total dose) we observed a significant tumour growth delay compared to the non-irradiated controls. This corroborates earlier findings showing exceptional *in vivo* radiation sensitivity of this glioblastoma cell line but is in contrast to the clinical experience of high radiation resistance in glioblastoma tumours [32-34].

The combination of fractionated irradiation with 8 parenteral injections of 40 mg/kg BW Erufosine 4 days before and during the fractionated irradiation was obviously able to intensify the radiation-induced decrease in tumour volume. The failure to detect more than borderline significant differences in the growth-delay of tumours treated with fractionated irradiation alone and fractionated irradiation plus Erufosine may at least partially be due to the unexpected high rate of local tumour control rates that were observed in response to fractionated irradiation alone in the combination experiment 1): Only a low rate of local controls (14%) had been observed in the dose-finding experiment in the highest dose-group (5 × 4.5 Gy) and no local controls had been observed in the 5 × 3.5 Gy dose-group. In contrast, a largely increased rate of local controls (28.6%) was observed in the combined treatment experiment (experiment 2) upon fractionated irradiation with a dose of 5 × 3.3 Gy. The reason for the discrepancy between the two experiments is unknown. Only 66% of transplanted animals developed subcutaneous T98G tumours which may be indicative of a residual antitumour immunoreactivity in the NMRI nu/nu mice [35]. But Krause and coworkers had already demonstrated in an earlier study that TCD50 values for T98G tumours do not differ in mice that received whole body irradiation prior to implantation of the xenograft tumours [34]. Therefore, we assume that a residual immune response of the host may not be causative for the differential effects observed in the two experiments.

Finally, when analyzing whether the addition of short-term Erufosine treatment to fractionated irradiation would improve tumour control probability by measuring the tumour control rates after additional top up irradiations we found that short-term Erufosine failed to improve the outcome of fractionated radiotherapy in terms of local tumour control. This suggests that although tumour regrowth was slightly retarded by co-treatment with erufosine no additional effect on the eradication of clonogenic tumour stem cells could be achieved.

Notably, the TCD50 value for T98G xenografts detected in the present study (12 Gy Top-up irradiation dose and a total irradiation dose of about 30 Gy under ambient conditions) was in the range of the TCD50 values reported in earlier studies for T98G xenografts after single dose or fractionated irradiation [33,34,36] although a comparison between the different studies remains difficult due to variations in the irradiation scheme (single/fractionated/mixed) and oxygenation conditions (ambient/hypoxic). In the present study, Top-up dose irradiations were administered under ambient conditions. Therefore, the lack of a difference in tumour control experiments in spite of a significant effect of Erufosine in growth delay experiments may be due to an increase of tumour hypoxia at the stem cell level induced by Erufosine during fractionated radiotherapy.

Up to now, only few studies are available that tested a potential benefit of APC in combination with radiotherapy *in vivo*. However, those studies were restricted to the use of the orally available APC Perifosine and the evaluation of tumour growth delay. Consistent with our observations with the novel intravenously applicable APC Erufosine, De la Pena et al. [22] observed a growth delay of U251 glioblastoma xenografts after repeated oral administrations of the closely related alkylphosphocholine Perifosine but also failed to detect an increased efficacy when Perifosine-treatment was combined with a single dose of 4 Gy at the onset of drug treatment. Given the comparable *in vivo* radiosensitivity of the two glioblastoma cell lines T89G and U251 [33] the irradiation dose used might be too low for a sustained combination effect, although a growth delay of single modality treatment was detectable.

In contrast, the same protocol of Perifosine-treatment combined with 2 × 5 Gy on days 2 and 4 caused a significant growth delay in a prostate cancer xenograft model compared to untreated controls whereas the single modality treatment was not effective [24]. Notably, even a sustained tumour regression was demonstrated after combined treatment with Perifosine and irradiation (5 Gy on days 2 and 4) in xenografts of squamous cell carcinoma [37]. Importantly, in this study effectiveness of Perifosine-treatment depended on the treatment duration and cumulative drug dose.

Also, the following limitations of the preclinical models have to be carefully considered: (i) *In vitro* radiosensitivity does not necessarily correlate to the tumour control probabilities determined *in vivo*[33]. (ii) Translation of findings from xenograft models to the clinical situation is even more difficult, particularly in the case of high-grade glioma, because of obvious differences in the tumour microenvironment and the growth behaviour of the malignant cells *in vivo*[38]. In this scenario, the xenograft model will underestimate additional modes of drug action, such as inhibition of migration and invasion and antiangiogenic effects [39-42]. However, up to now the use of more appropriate preclinical models for the evaluation of the efficacy of combined treatment approaches with radiotherapy *in vivo*, e.g. orthotopic or spontaneous tumours, is still sparse because of the limited availablity of image-guided radiation systems for small animals.

In conclusion, the intervenously applicable APC Erufosine with proven ability to cross the blood brain barrier causes a transient decrease in the growth of T98G glioblastoma tumours *in vivo* but fails to improve efficacy of fractionated irradiation in terms of local tumour control. Further *in vivo* studies are needed to evaluate whether extended Erufosine treatment may be more effective in terms of radiosensitization and whether the drug may interfere with the known adverse biological factors that limit the efficacy of radiotherapy *in vivo.*

## Competing interests

The authors declare that they have no competing intereststhat could inappropriately influence this work.

## Authors' contributions

GH contributed significantly to the design of the study, data acquisition, data analysis and drafting the manuscript. VM contributed significantly to data acquisition, data analysis and drafting the manuscript. LHL contributed significantly to data acquisition and data analysis. HE synthesized and provided ErPC and ErPC3 for the analysis. MB performed critical revision of the manuscript. CB participated in the conception of the study and interpretation of data. WB substantially contributed to evaluation and interpretation of data and performed critical revision of the manuscript. VJ performed conception and design of the study and substantially contributed to interpretation of data, drafting of the manuscript, critical revision of the manuscript and final approval. All authors read and approved the final manuscript.
